# Are mind-body therapies beneficial for older people with dementia? A Systematic Review and meta-analysis of randomized controlled trials

**DOI:** 10.3389/fpsyt.2025.1569709

**Published:** 2025-04-14

**Authors:** Zhengyang Mei, Chenyi Cai, Tingfeng Wang, Yuanzhuo Zhang, Wen Zhao, Chifong Lam, Shulai Luo, Yu Shi, Shi Luo

**Affiliations:** ^1^ School of Physical Education, Southwest University, Chongqing, China; ^2^ School of Physical Education, Shanghai University of Sport, Shanghai, China; ^3^ Faculty of Education, Southwest University, Chongqing, China; ^4^ Key Laboratory of Cognition and Personality, Faculty of Psychology, Ministry of Education, Southwest University, Chongqing, China

**Keywords:** mind-body therapies, older people, dementia, mental health, gerontology

## Abstract

**Objective:**

This systematic review and meta-analysis aimed to evaluate the potential multidomain benefits of mind-body therapies (MBTs) for behavioral and psychological symptoms of dementia (BPSD) in older people with dementia (OPWD).

**Methods:**

Relevant randomized controlled trials (RCTs) were identified using electronic databases and manual searches. Two independent researchers evaluated the risk of bias in the included trials using the Revised Cochrane Risk-of-Bias tool for randomized trials. A standardized mean difference (SMD) with a 95% confidence interval (CI) was used to combine effect sizes.

**Results:**

This review included 35 RCTs comprising 4,043 patients, of whom 24 were included in the meta-analyses. MBTs effectively improved BPSD (SMD = -0.33; 95% CI -0.49 to -0.16; *p* < 0.01), anxiety (SMD = -0.82; 95% CI -1.53 to -0.10; *p* = 0.02), and depression (SMD = -0.57; 95% CI -1.06 to -0.08; *p* = 0.02), with no significant improvements observed in agitation (SMD = -0.09; 95% CI -0.25 to 0.07; *p* = 0.27) among patients with dementia. The certainty of evidence across the outcomes ranged from low to very low, based on the Grading of Recommendations, Assessment, Development, and Evaluations ratings.

**Conclusion:**

Effective nursing for patients with dementia is vital, as they are undergoing a major transition in their physical and mental health. In clinical practice, healthcare and social care therapists should develop personalized intervention programs based on patient individual differences and the actual dose-response relationship, which will help maximize the clinical benefits of non-pharmacological treatments in the context of limited medical resources. More high-quality RCTs could be conducted to compare the differential efficacy of non-pharmacological treatments on various aspects of BPSD in OPWD to provide a better evidence base to guide individual care and policy guidance.

**Systematic review registration:**

PROSPERO, identifier CRD42024559809.

## 1 Introduction

Dementia is a progressive cognitive disorder that impairs daily functioning and is a primary contributor to dependency, disability, and death (1, 2). It is characterized by cognitive decline, deteriorating functional status, and worsening of neuropsychiatric symptoms (3). Currently, over 50 million individuals worldwide are affected by dementia, and projections indicate this figure could rise to 131 million by 2050 (3, 4). Moreover, the overall prevalence of dementia, from all causes, stands at 7.0%, with its rate of occurrence increasing sharply with age for both men and women (5), suggesting the growing public health challenge posed by dementia as the global population continues to age.

Old age is not only a critical stage for changes in physical and mental health but also a vulnerable period for the development of dementia. During this period, older people are often exposed to multiple risk factors for developing dementia, which are classified as immutable (age, sex, ethnicity, etc.) or potentially modifiable (diabetes, smoking, social isolation, physical inactivity, etc.) (6, 7). Moreover, compelling evidence suggests that dementia is prevalent among older people and may increase their susceptibility to physical and mental health problems, including cognitive impairment (8), suicidal behavior (9), epilepsy (10), disability (11), anxiety (12), and depression (13).

Dementia can be diagnosed based on medical history, cognitive and physical examination, laboratory testing, and brain imaging (3). As dementia is a progressive, incurable illness, several medications have been developed to help older people with dementia (OPWD) alleviate the physical and mental discomfort, including donepezil, cholinesterase inhibitors, memantine, and statins (14, 15). However, prolonged use of these drugs can result in numerous adverse outcomes. Specifically, the increasing use of cholinesterase inhibitors results in increased adverse drug reactions, such as cardiovascular and gastrointestinal adverse effects (16); treatment with antipsychotics in older people is associated with an increased risk of cardiovascular incidents and mortality (17). Therefore, no compelling evidence supports the use of these drugs in such patients (14).

In this regard, complementary therapies could offer potential solutions to these challenges. Mind-body therapies (MBTs), rooted in ancient Eastern practices, are a non-pharmacological approach that focuses on interactions between mind, body, and spirit to improve mental and physical well-being (18, 19). Compared with other medications, MBTs are characterized by low risk and high therapeutic benefits and can be alternatives or adjuvant approaches to conventional treatments (20, 21). Common MBTs include Mindfulness training, Tai Chi, Qigong, Baduanjin, Yoga, Pilates, and Music therapy, which have been proven to exert beneficial effects on the physical and mental health of OPWD (22–28). However, evidence regarding the efficacy of MBTs in managing the behavioral and psychological symptoms of dementia (BPSD) in OPWD is inconsistent, and some studies do not support the significant efficacy of MBTs in this area (29–33). This study aimed to evaluate the potential multidomain benefits of MBTs for BPSD in OPWD.

## 2 Methods

This systematic review and meta-analysis adhered to the Preferred Reporting Items for Systematic Reviews and Meta-Analyses (PRISMA) 2020 (34) and was registered in the International Prospective Register of Systematic Reviews (PROSPERO) under the registration number CRD42024559809.

### 2.1 Search methods

Using Medical Subject Headings in conjunction with Boolean and proximity operators, a search was conducted across six electronic databases: PubMed, Embase, Web of Science, Scopus, EBSCOhost, and APA PsycINFO, to identify relevant literature. The search covered the period from the inception of each database up to June 2024, and the strategy adhered to the Population, Intervention, Comparator, Outcome, and Study design framework. Details of the search strategy are outlined in **Table 1**, as per the PubMed database.

**Table 1:** PubMed search strategy.

#1	Dementia* [MeSH Terms]
#2	Dementia* [Title/Abstract] OR Alzheimer* [Title/Abstract] OR Amentia* [Title/Abstract]
#3	#1 OR #2
#4	Mind body* [Title/Abstract] OR Mind-body* [Title/Abstract] OR Mindfulness [Title/Abstract] OR Meditation [Title/Abstract] OR Shadow boxing [Title/Abstract] OR Tai Ji [Title/Abstract] OR Tai-ji [Title/Abstract] OR Tai Chi [Title/Abstract] OR Chi, Tai [Title/Abstract] OR Tai Ji Quan [Title/Abstract] OR Ji Quan, Tai [Title/Abstract] OR Quan, Tai Ji [Title/Abstract] OR Taiji [Title/Abstract] OR Taijiquan [Title/Abstract] OR T’ai Chi [Title/Abstract] OR Tai Chi Chuan [Title/Abstract] OR Qigong [Title/Abstract] OR Qi Gong [Title/Abstract] OR Ch’i Kung [Title/Abstract] OR Baduanjin [Title/Abstract] OR Yoga [Title/Abstract] OR Pilates [Title/Abstract] OR Exercise Movement Techniques [Title/Abstract] OR Movement Techniques, Exercise [Title/Abstract] OR Exercise Movement Technics [Title/Abstract] OR Pilates-Based Exercises [Title/Abstract] OR Exercises, Pilates-Based [Title/Abstract] OR Pilates Based Exercises [Title/Abstract] OR Pilates Training [Title/Abstract] OR Training, Pilates [Title/Abstract] OR Music* [Title/Abstract]
#5	Older* [Title/Abstract] OR Elder* [Title/Abstract] OR Senior* [Title/Abstract] OR Aged [Title/Abstract]
#6	Randomized controlled trial [Publication Type] OR Randomized [Title/Abstract] OR Placebo [Title/Abstract]
#7	#3 AND #4 AND #5 AND #6

### 2.2 Inclusion and exclusion criteria

The criteria for inclusion and exclusion of studies are presented in **Table 2**.

**Table 2:** Inclusion and exclusion criteria.

Category	Inclusion criteria	Exclusion criteria
Population	Older people with dementia (age 60 years or older)	Not older people with dementia
Intervention	Mind-body therapies were used as the generic term for interventions including Mindfulness training, Tai Chi, Qigong, Baduanjin, Yoga, Pilates, Music therapy, etc	Interventions that were not mind-body therapies
Comparator	Control group receiving only routine treatment or appropriate rehabilitation intervention	No exclusion criteria
Outcome	Any assessment for behavioral and psychological symptoms of dementia	No exclusion criteria
Study design	All types of randomized controlled trials	Non-randomized controlled trials, such as uncontrolled before-after trials, quasi-experiments, literature review, study protocols, conference proceedings, comments, editorial, letter to editors, etc

### 2.3 Study selection and quality appraisal

Two independent researchers, following predefined inclusion and exclusion criteria, utilized EndNote 20.6 for study selection. After duplicates were eliminated, the remaining references were independently reviewed by both researchers based on titles, abstracts, and full texts. Quality appraisal of the included trials was conducted using the Revised Cochrane risk-of-bias tool for randomized trials, with evaluation across five bias domains: (a) randomization process, (b) deviations from intended interventions, (c) missing outcome data, (d) measurement of the outcome, and (e) selection of the reported result (35). Disagreements during study selection and quality appraisal processes were resolved through consultation with a third author.

### 2.4 Data extraction

Two independent researchers used a data extraction form to gather relevant information from each included trial. The extracted data comprised the following: (1) basic details such as the first author, country, and year of publication; (2) participant characteristics, including mean age (with standard deviation), sample size, and the percentage of male participants; (3) diagnostic criteria for dementia; (4) intervention and control conditions; and (5) outcome and measure.

### 2.5 Data synthesis

Given the differences in the measurement scales used for continuous variables (BPSD, anxiety, depression, and agitation) across the included trials, standardized mean differences (SMDs; Cohen’s d) with 95% confidence intervals (CIs) for each outcome were pooled and presented in forest plots using Stata software, version 18.0 (36). Statistical heterogeneity between trials was assessed using the chi-square test based on *Q*-test and *I^2^* statistics, with a significance threshold of *p*-value < 0.10 (37). A sensitivity analysis was performed for each outcome using a stepwise elimination method to assess whether the pooled results were significantly affected by individual studies (36). Given the number of included trials, publication bias was examined by visual inspection of funnel plots and using Egger’s test (38, 39). The trim-and-fill method was used to assess the robustness of the findings in the presence of a publication bias (40). The certainty of evidence for each outcome was evaluated by two independent researchers using the Grading of Recommendations, Assessment, Development, and Evaluations (GRADE) guidelines (41). All statistical analyses were performed using Stata 18.0.

## 3 Results

### 3.1 Search outcomes

**Figure 1:**
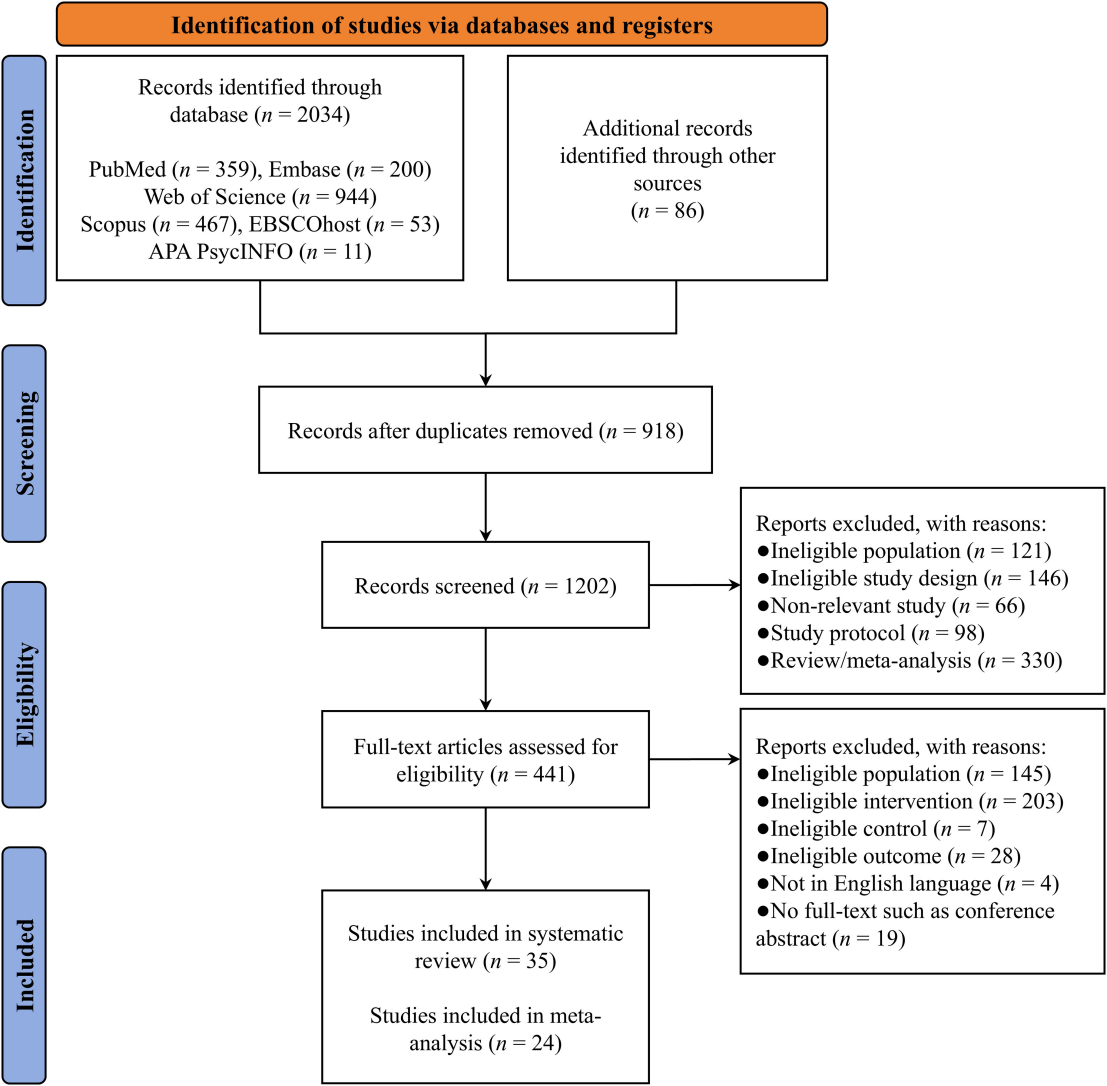
PRISMA flow diagram.

Searches of electronic databases and additional sources yielded 2,120 results, of which 918 were duplicates. After the titles and abstracts of the remaining 1,202 records were screened, the full text of 441 articles was assessed, resulting in 35 eligible randomized controlled trials (RCTs) (42–76). After 11 additional trials with missing quantitative data were excluded, 24 were included in the meta-analyses. A PRISMA flow diagram of the literature search is presented in **Figure 1**.

### 3.2 Study characteristics

The 35 included trials (see **Table 3**) were published between 2006 and 2024 and were conducted in various countries, including China (13 trials), Italy (6 trials), the United States (4 trials), Australia (3 trials), Germany (2 trials), the Netherlands (2 trials), the United Kingdom (2 trials), Denmark, France, and Japan (one trial each). In total, 2,065 patients were assigned to the experimental group, with a mean age ranging from 76.78 to 86.56 years, while 1,978 patients were assigned to the control group, with a mean age of 76.80 to 87.20 years.

**Table 3:** Main characteristics of the included randomized controlled trials.

Study ID	Country	Population	Age (Mean (SD))	Total/M%	Diagnostic criteria	Intervention	Control	Outcome (measure)
Baker et al., 2022 (42)	Australia	OPWD	T: 86.00 (7.50) C: 87.20 (6.50)	T: 45/NR C: 50/NR	CDR/MMSE	Group music	SC	Depression (MADRS) BPSD (NPI)
Ceccato et al., 2012 (43)	Italy	OPWD	T: 85.50 (5.90) C: 87.20 (7.10)	T: 27/22.2% C: 23/17.4%	DSM-IV	Traditional music	SC	Depression (GDS) Agitation (CMAI)
Chen and Pei, 2018 (44)	China	OPWD	T: 77.30 (9.40) C: 77.30 (10.00)	T: 15/40.0% C: 13/61.5%	Medical diagnosis/MMSE/CDR	Traditional music	TAU	Agitation (CMAI)
Cheng et al., 2012 (45)	China	OPWD	T: 81.00 (7.70) C: 82.50 (7.10)	T: 12/50.0% C: 12/25.0%	MMSE/CDR	Tai Chi	TAU	Depression (GDS)
Cheung et al., 2020 (46)	China	OPWD	T: 85.71 (6.68) C: 85.58 (7.46)	T: 58/25.9% C: 53/24.5%	Medical diagnosis/Global Deterioration Scale	Music with movement	TAU	Agitation (CMAI)
Chu et al., 2014 (47)	China	OPWD	T+C: 82.00 (6.80)	T: 49/NR C: 51/NR	DSM-IV	Group music	TAU	Depression (CSDD)
Churcher Clarke et al., 2017 (66)	UK	OPWD	T: 81.30 (9.29) C: 79.36 (9.91)	T: 20/40.0% C: 11/72.7%	Medical diagnosis/MMSE/DSM-IV	Mindfulness	TAU	Anxiety (RAID) Depression (CSDD)
Cooke et al., 2010a (48)	Australia	OPWD	T: NR C: NR	T: 24/NR C: 23/NR	Medical diagnosis/MMSE/DSM-IV	Group music	TAU	Depression (GDS)
Cooke et al., 2010b (49)	Australia	OPWD	T: NR C: NR	T: 24/NR C: 23/NR	Medical diagnosis/MMSE/DSM-IV	Group music	TAU	Anxiety (RAID) Agitation (CMAI)
Giulietti et al., 2023 (67)	Italy	OPWD	T: 82.80 (5.60) C: 82.90 (4.20)	T: 22/36.4% C: 22/22.7%	NINCDS-ADRDA	Mindfulness	No-intervention	Depression (BDI)
Guétin et al., 2009 (50)	France	OPWD	T: 85.20 (6.00) C: 86.90 (5.20)	T: 14/NR C: 12/NR	Medical diagnosis	Personalized music	TAU	Anxiety (HAMA) Depression (GDS)
Harrison et al., 2021 (68)	USA	OPWD	T: 79.70 (11.20) C: 80.60 (12.60)	T: 103/28.4% C: 55/38.0%	Medical diagnosis	Personalized music	TAU	Agitation (CMAI)
Hillebrand et al., 2023 (51)	Germany	OPWD	T: 83.84 (7.25) C: 84.00 (6.69)	T: 44/18.2% C: 46/26.1%	Medical diagnosis	Personalized music	TAU	BPSD (DeCS)
Huang et al., 2019 (52)	China	OPWD	T: 81.90 (6.00) C: 81.90 (6.10)	T: 36/NR C: 38/NR	DSM-IV/CDR	Tai Chi	TAU	Depression (GDS) BPSD (NPI)
Lin et al., 2011 (53)	China	OPWD	T: 81.46 (7.34) C: 82.15 (6.28)	T: 49/46.9% C: 51/47.1%	DSM-IV	Group music	TAU	Agitation (CMAI)
Liu et al., 2021 (54)	China	OPWD	T: 86.56 (4.54) C: 86.92 (5.73)	T: 25/NR C: 25/NR	CDR	Music with movement	TAU	Anxiety (HAMA) Depression (GDS)
Lyu et al., 2018 (55)	China	OPWD	T: 68.90 (7.10) C: 69.90 (7.90)	T: 97/41.2% C: 95/41.1%	NINCDS-ADRDA	Traditional music	TAU	BPSD (NPI)
McCreedy et al., 2022 (69)	USA	OPWD	T: 79.80 (12.20) C: 80.80 (12.10)	T: 483/32.3% C: 493/29.2%	Medical diagnosis	Personalized music	TAU	Agitation (CMAI)
Noone et al., 2023 (70)	UK	OPWD	T: 77.80 (10.63) C: 76.80 (4.96)	T: 10/10.0% C: 10/40.0%	DSM-IV/MMSE/PHQ	Mindfulness	TAU	Anxiety (RAID) Depression (CSDD)
Park et al., 2020 (56)	USA	OPWD	T+C: 84.30 (7.70)	T: 10/NR C: 10/NR	Medical diagnosis/MMSE	Yoga	TAU	Anxiety (HADS) Depression (HADS) Agitation (CMAI)
Prick et al., 2024 (71)	Netherlands	OPWD	T: 81.70 (7.60) C: 82.30 (9.90)	T: 49/38.8% C: 53/45.3%	Medical diagnosis	Personalized music	TAU	BPSD (NPI)
Raglio et al., 2015 (57)	Italy	OPWD	T: 81.70 (7.80) C: 82.40 (6.80)	T: 32/NR C: 35/NR	DSM-IV/CDR/MMSE/NPI	Personalized music	SC	BPSD (NPI)
Raglio et al., 2010a (58)	Italy	OPWD	T: 85.40 (6.50) C: 84.60 (6.80)	T: 30/3.3% C: 30/13.3%	Medical diagnosis/MMSE/DSM-IV	Traditional music	SC	BPSD (NPI)
Raglio et al., 2010b (72)	Italy	OPWD	T: 84.00 (6.00) C: 87.00 (6.00)	T: 10/20.0% C: 10/30.0%	NINCDS-ADRDA NINDS-AIREN	Traditional music	SC	BPSD (NPI)
Raglio et al., 2008 (73)	Italy	OPWD	T: 84.40 (5.50) C: 85.80 (5.40)	T: 30/16.6% C: 29/13.8%	DSM-IV/MMSE/CDR	Traditional music	SC	BPSD (NPI)
Ridder et al., 2013 (59)	Denmark	OPWD	T: 82.17 (8.84) C: 80.20 (8.67)	T: 20/NR C: 21/NR	Medical diagnosis/MMSE/Global Deterioration Scale	Personalized music	SC	Agitation (CMAI)
Sakamoto et al., 2013 (60)	Japan	OPWD	T: 80.40 (7.41) C: 81.54 (7.82)	T: 13/15.4% C: 13/15.4%	DSM-IV/MMSE/CDR	Personalized music	TAU	BPSD (BEHAVE-AD)
Sing et al., 2019 (61)	China	OPWD	T: 85.00 (7.10) C: 85.70 (7.00)	T: 40/30.0% C: 33/30.3%	Medical diagnosis	Group music	SC	BPSD (NPI)
Sisti et al., 2024 (74)	USA	OPWD	T: 79.80 (12.20) C: 80.80 (12.10)	T: 483/32.3% C: 493/29.2%	Medical diagnosis	Personalized music	TAU	BPSD (ABMI)
Sung et al., 2006a (62)	China	OPWD	T: NR C: NR	T: 32/NR C: 25/NR	Medical diagnosis	Personalized music	TAU	Agitation (CMAI)
Sung et al., 2006b (63)	China	OPWD	T: 76.78 (9.12) C: 78.44 (7.85)	T: 18/61.1% C: 18/83.3%	DSM-IV/Global Deterioration Scale	Music with movement	TAU	Agitation (CMAI)
Sung et al., 2012 (64)	China	OPWD	T: 81.37 (9.14) C: 79.50 (8.76)	T: 27/NR C: 28/NR	Medical diagnosis	Group music	TAU	Anxiety (RAID) Agitation (CMAI)
Vink et al., 2013 (75)	Netherlands	OPWD	T: 82.42 (7.62) C: 81.76 (5.72)	T: 43/32.6% C: 34/26.5%	DSM-IV	Group music	TAU	Agitation (CMAI)
Weise et al., 2020 (76)	Germany	OPWD	T+C: 85.05 (5.93)	T: 10/NR C: 10/NR	Medical diagnosis	Personalized music	Wait-list	Agitation (CMAI)
Xu et al., 2024 (65)	China	OPWD	T: NR C: NR	T: 61/52.5% C: 60/55.0%	Medical diagnosis	Group music	TAU	Depression (CSDD)

T, Test group; C, Control group; M%, Percentage of males; OPWD, Older People with Dementia; TAU, Treatment as usual; SC, Standard care; ABMI, Agitated Behavior Mapping Instrument; BPSD, Behavioral and Psychological Symptoms of Dementia; BDI, Beck Depression Inventory; BEHAVE-AD, Behavioral Pathology in Alzheimer’s Disease Rating Scale; CDR, Clinical Dementia Rating; CSDD, Cornell Scale for Depression in Dementia; CMAI, Cohen-Mansfield Agitation Inventory; DSM-IV, Diagnostic and Statistical Manual of Mental Disorders-IV; DeCS, Dementia Coding System; GDS, Geriatric Depression Scale; HAMA, Hamilton Anxiety Rating Scale; HADS, Hospital Anxiety and Depression Scale; MMSE, Mini-Mental State Examination; MADRS, Montgomery-Asberg Depression Rating Scale; NPI, Neuropsychiatric Inventory; NINCDS-ADRDA, National Institute of Neurological and Communicative Disorders and Stroke-Alzheimer’s Disease and Related Disorders Association; NINDS-AIREN, National Institute of Neurological Disorders and Stroke-Association Internationale pour la Recherche et l’Enseignement en Neurosciences; PHQ, Patient Health Questionnaire; RAID, Rating Anxiety in Dementia Scale.

Among the 35 included trials, the diagnostic criteria for dementia primarily consisted of medical diagnosis, Diagnostic and Statistical Manual of Mental Disorders-IV, Mini-Mental State Examination, Clinical Dementia Rating, and the Global Deterioration Scale. The interventions included personalized music (11 trials), group music (9 trials), traditional music (6 trials), mindfulness (3 trials), music with movement (3 trials), Tai Chi (2 trials), and yoga (1 trial). The controls included treatment as usual (25 trials), standard care (8 trials), no-intervention (1 trial), and wait-list (1 trial). In addition, the deliverers of the intervention primarily included music therapists, musicians, nursing staff, psychotherapist, and trained facilitators, all of whom were qualified to be responsible for the implementation of the intervention.

### 3.3 Quality appraisal

**Figure 2:**
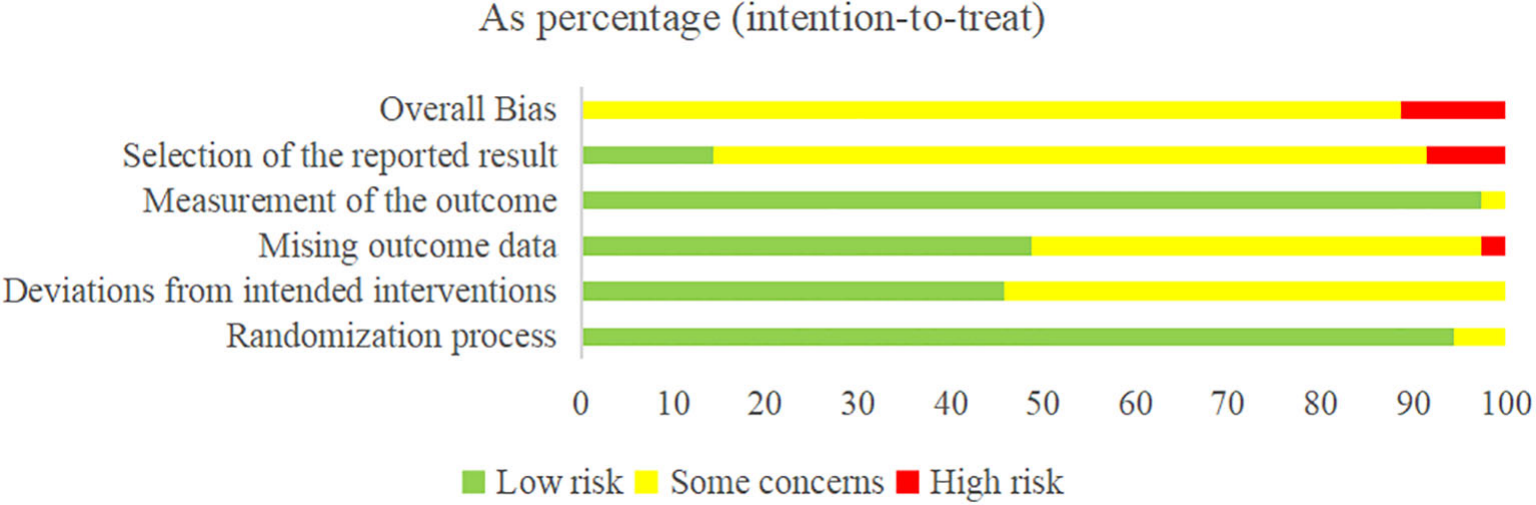
Risk-of-bias summary.

The risk of bias ranged from some concern to high (see **Figure 2**, **Table 4**). The main flaws (≥10% high risk) across the included trials were the randomization process (94.3% low risk, 5.7% some concerns, and 0.0% high risk); deviations from intended interventions (45.7% low risk, 54.3% some concerns, and 0.0% high risk); missing outcome data (48.6% low risk, 48.6% some concerns, and 2.8% high risk); outcome measurement (97.2% low risk, 2.8% some concerns, and 0.0% high risk); and selection of the reported results (14.3% low risk, 77.1% some concerns, and 8.6% high risk).

**Table 4:** Risk of bias summary for the included effect estimates.

Study ID	Outcome	Domains					
		D1	D2	D3	D4	D5	Overall rating
Baker et al., 2022 (42)	Depression	Low	Low	Some concerns	Low	Some concerns	Some concerns
Baker et al., 2022 (42)	BPSD	Low	Low	Some concerns	Low	Some concerns	Some concerns
Ceccato et al., 2012 (43)	Depression	Low	Some concerns	Low	Low	Low	Some concerns
Ceccato et al., 2012 (43)	Agitation	Low	Some concerns	Low	Low	Low	Some concerns
Chen and Pei, 2018 (44)	Agitation	Low	Low	Some concerns	Low	Low	Some concerns
Cheng et al., 2012 (45)	Depression	Low	Some concerns	Low	Low	Some concerns	Some concerns
Cheung et al., 2020 (46)	Agitation	Low	Low	Low	Low	Some concerns	Some concerns
Chu et al., 2014 (47)	Depression	Low	Low	Low	Low	Some concerns	Some concerns
Churcher Clarke et al., 2017 (66)	Anxiety	Low	Some concerns	Some concerns	Low	Some concerns	Some concerns
Churcher Clarke et al., 2017 (66)	Depression	Low	Some concerns	Some concerns	Low	Some concerns	Some concerns
Cooke et al., 2010a (48)	Depression	Low	Low	Some concerns	Low	Some concerns	Some concerns
Cooke et al., 2010b (49)	Anxiety	Low	Low	Some concerns	Low	Some concerns	Some concerns
Cooke et al., 2010b (49)	Agitation	Low	Low	Some concerns	Low	Some concerns	Some concerns
Giulietti et al., 2023 (67)	Depression	Low	Some concerns	Low	Low	Some concerns	Some concerns
Guétin et al., 2009 (50)	Anxiety	Low	Low	Some concerns	Low	Some concerns	Some concerns
Guétin et al., 2009 (50)	Depression	Low	Low	Some concerns	Low	Some concerns	Some concerns
Harrison et al., 2021 (68)	Agitation	Low	Some concerns	Some concerns	Low	Some concerns	Some concerns
Hillebrand et al., 2023 (51)	BPSD	Low	Some concerns	High	Some concerns	Low	High
Huang et al., 2019 (52)	Depression	Low	Some concerns	Some concerns	Low	Some concerns	Some concerns
Huang et al., 2019 (52)	BPSD	Low	Some concerns	Some concerns	Low	Some concerns	Some concerns
Lin et al., 2011 (53)	Agitation	Low	Some concerns	Low	Low	Some concerns	Some concerns
Liu et al., 2021 (54)	Anxiety	Low	Some concerns	Low	Low	Some concerns	Some concerns
Liu et al., 2021 (54)	Depression	Low	Some concerns	Low	Low	Some concerns	Some concerns
Lyu et al., 2018 (55)	BPSD	Low	Some concerns	Low	Low	Some concerns	Some concerns
McCreedy et al., 2022 (69)	Agitation	Low	Low	Some concerns	Low	Some concerns	Some concerns
Noone et al., 2023 (70)	Anxiety	Low	Low	Some concerns	Low	Some concerns	Some concerns
Noone et al., 2023 (70)	Depression	Low	Low	Some concerns	Low	Some concerns	Some concerns
Park et al., 2020 (56)	Anxiety	Low	Low	Some concerns	Low	Some concerns	Some concerns
Park et al., 2020 (56)	Depression	Low	Low	Some concerns	Low	Some concerns	Some concerns
Park et al., 2020 (56)	Agitation	Low	Low	Some concerns	Low	Some concerns	Some concerns
Prick et al., 2024 (71)	BPSD	Low	Low	Low	Low	High	High
Raglio et al., 2015 (57)	BPSD	Low	Low	Some concerns	Low	Some concerns	Some concerns
Raglio et al., 2010a (58)	BPSD	Low	Some concerns	Some concerns	Low	High	High
Raglio et al., 2010b (72)	BPSD	Low	Some concerns	Low	Low	Low	Some concerns
Raglio et al., 2008 (73)	BPSD	Low	Some concerns	Some concerns	Low	High	High
Ridder et al., 2013 (59)	Agitation	Low	Low	Low	Low	Some concerns	Some concerns
Sakamoto et al., 2013 (60)	BPSD	Low	Some concerns	Low	Low	Some concerns	Some concerns
Sing et al., 2019 (61)	BPSD	Some concerns	Low	Some concerns	Low	Some concerns	Some concerns
Sisti et al., 2024 (74)	BPSD	Low	Low	Some concerns	Low	Some concerns	Some concerns
Sung et al., 2006a (62)	Agitation	Low	Some concerns	Low	Low	Low	Some concerns
Sung et al., 2006b (63)	Agitation	Low	Some concerns	Low	Low	Some concerns	Some concerns
Sung et al., 2012 (64)	Anxiety	Low	Some concerns	Low	Low	Some concerns	Some concerns
Sung et al., 2012 (64)	Agitation	Low	Some concerns	Low	Low	Some concerns	Some concerns
Vink et al., 2013 (75)	Agitation	Low	Low	Some concerns	Low	Some concerns	Some concerns
Weise et al., 2020 (76)	Agitation	Some concerns	Some concerns	Low	Low	Some concerns	Some concerns
Xu et al., 2024 (65)	Depression	Low	Some concerns	Low	Low	Some concerns	Some concerns

BPSD, Behavioral and Psychological Symptoms of Dementia; D1, Randomization process; D2, Deviations from intended interventions; D3, Missing outcome data; D4, Measurement of the outcome; D5, Selection of the reported result.

Green for "low risk," yellow for "some concerns," and red for "high risk".

### 3.4 Pairwise meta-analyses

#### 3.4.1 Effects of MBTs on BPSD

**Figure 3:**
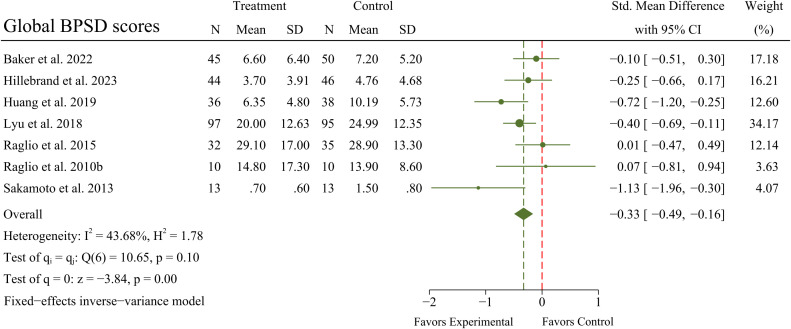
Main analyses for the effects of MBTs on BPSD.

A meta-analysis of 7 RCTs (N = 564 patients) exhibited a significant improvement in BPSD (SMD = -0.33; 95% CI -0.49 to -0.16; *p* < 0.01) in OPWD, with no significant heterogeneity between RCTs (*I^2^* = 43.68%; *Q* = 10.65; *p* = 0.10). A forest plot for BPSD is presented in **Figure 3**.

#### 3.4.2 Effects of MBTs on anxiety

**Figure 4:**
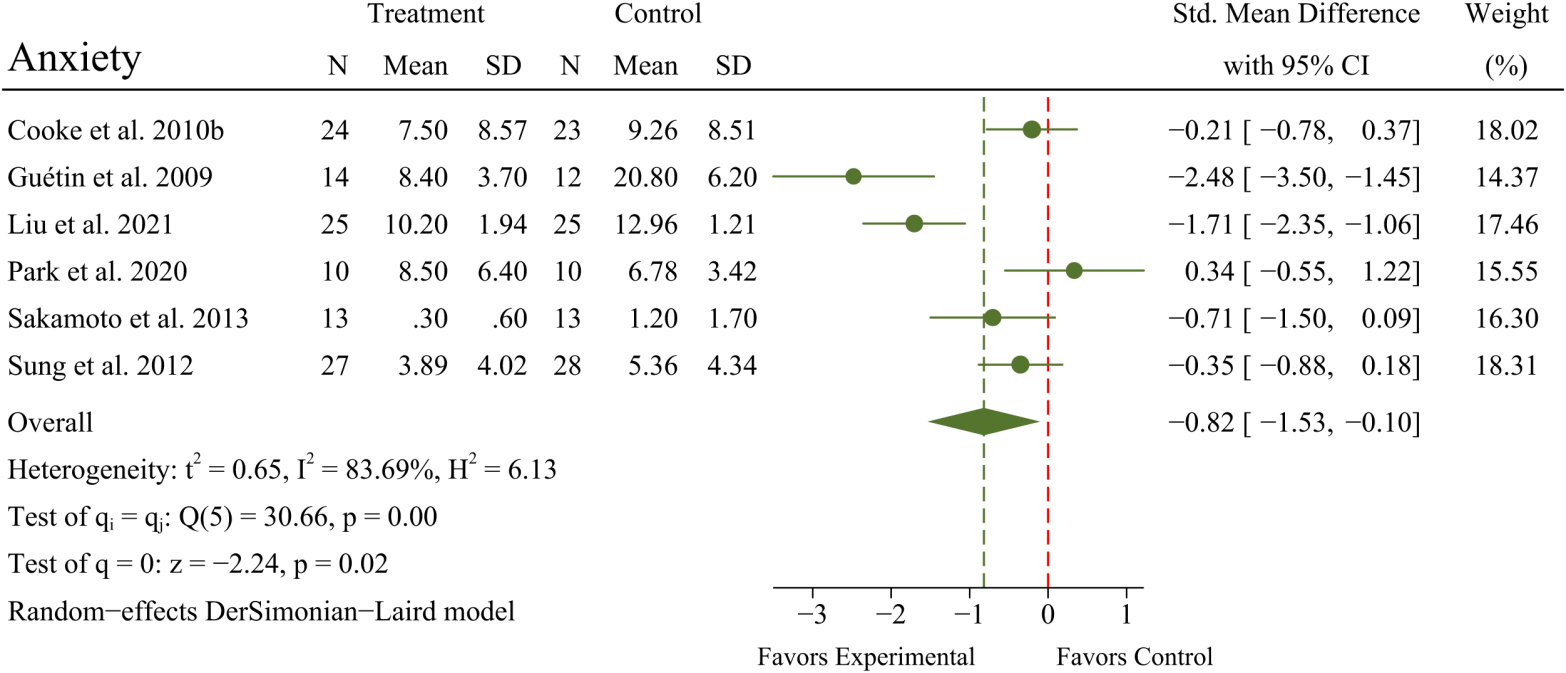
Main analyses for the effects of MBTs on anxiety.

A meta-analysis of 6 RCTs (N = 224 patients) exhibited a significant improvement in anxiety (SMD = -0.82; 95% CI -1.53 to -0.10; *p* = 0.02) in OPWD, but with significant heterogeneity between RCTs (*I^2^* = 83.69%; *Q* = 30.66; *p* < 0.01). A forest plot for anxiety is presented in **Figure 4**.

#### 3.4.3 Effects of MBTs on depression

**Figure 5:**
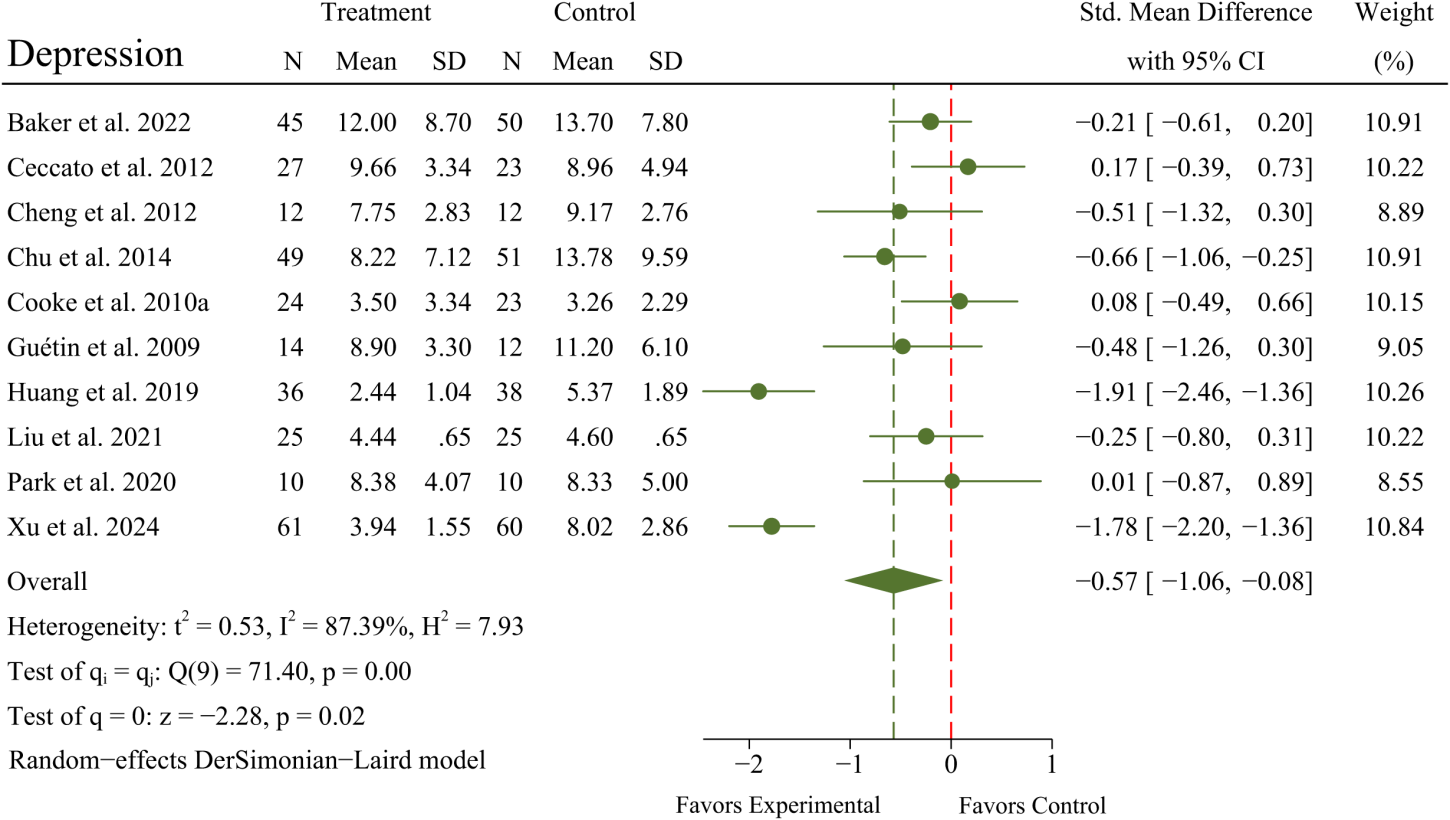
Main analyses for the effects of MBTs on depression.

A meta-analysis of 10 RCTs (N = 607 patients) exhibited a significant improvement in depression (SMD = -0.57; 95% CI -1.06 to -0.08; *p* = 0.02) in OPWD, but with significant heterogeneity between RCTs (*I^2^* = 87.39%; *Q* = 71.40; *p* < 0.01). A forest plot for depression is presented in **Figure 5**.

#### 3.4.4 Effects of MBTs on agitation

**Figure 6:**
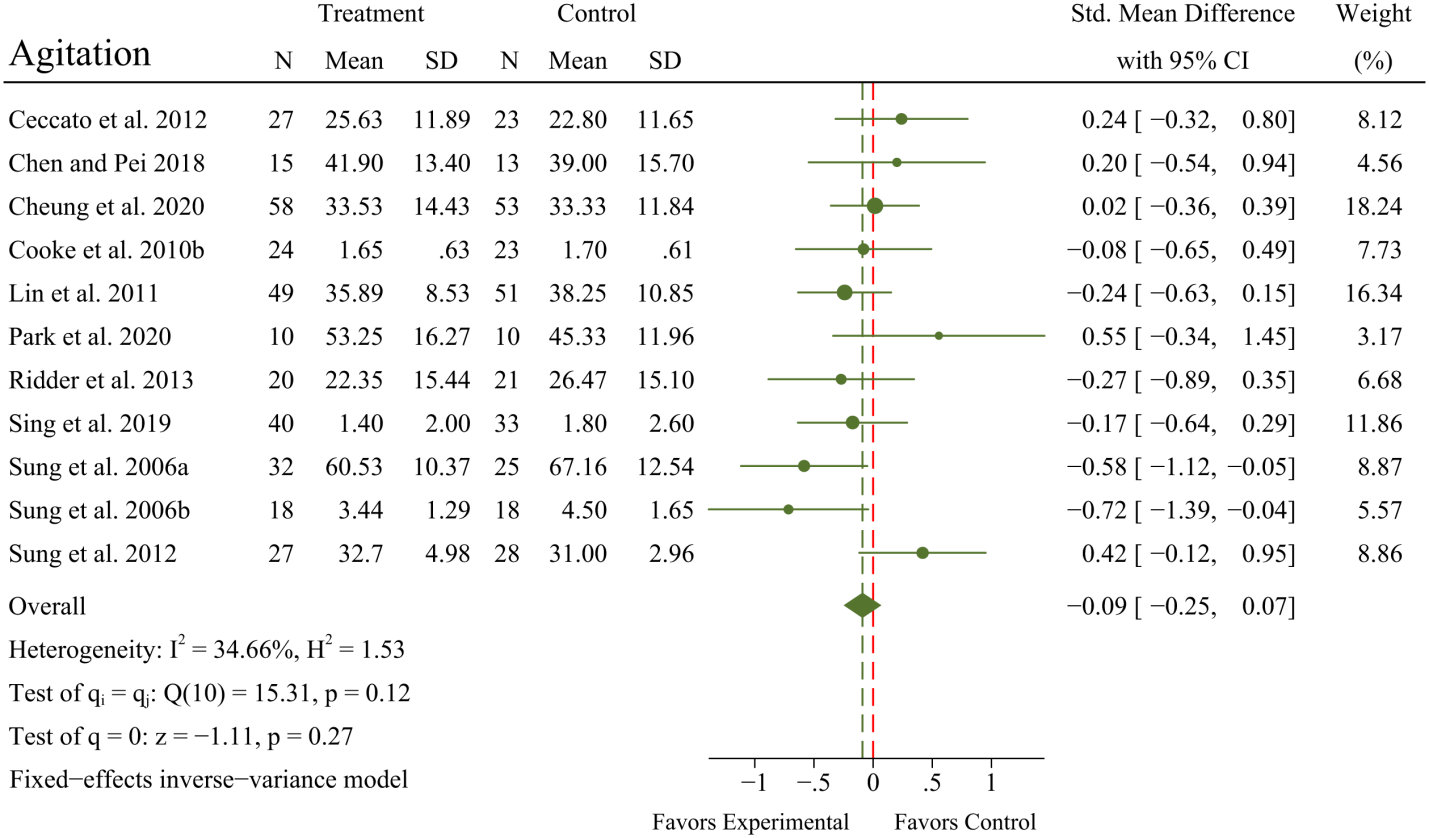
Main analyses for the effects of MBTs on agitation.

A meta-analysis of 11 RCTs (N = 618 patients) exhibited no significant improvement in agitation (SMD = -0.09; 95% CI -0.25 to 0.07; *p* = 0.27) in OPWD, with no significant heterogeneity between RCTs (*I^2^* = 34.66%; *Q* = 15.31; *p* = 0.12). A forest plot for agitation is presented in **Figure 6**.

### 3.5 Sensitivity analyses

The results of the sensitivity analyses indicated that the pooled results for agitation and BPSD remained stable after excluding individual studies, indicating that these results were robust and insensitive to study selection. However, the pooled results for anxiety and depression were sensitive to study selection and were less robust when individual studies were excluded. The results of the sensitivity analyses are presented in **Table 5**.

**Table 5:** Sensitivity analyses for outcomes by omitting individual studies.

Outcome	Study omitted	SMD	95% CI	
			Lower bound	Upper bound
BPSD	Baker et al., 2022 (42)	-0.37	-0.56	-0.19
	Hillebrand et al., 2023 (51)	-0.34	-0.53	-0.16
	Huang et al., 2019 (52)	-0.27	-0.45	-0.09
	Lyu et al., 2018 (55)	-0.29	-0.50	-0.08
	Raglio et al., 2015 (57)	-0.37	-0.55	-0.20
	Raglio et al., 2010b (72)	-0.34	-0.51	-0.17
	Sakamoto et al., 2013 (60)	-0.29	-0.46	-0.12
Anxiety	Cooke et al., 2010b (49)	-0.95	-1.81	-0.09
	Guétin et al., 2009 (50)	-0.54	-1.18	0.09
	Liu et al., 2021 (54)	-0.62	-1.32	0.09
	Park et al., 2020 (56)	-1.02	-1.78	-0.27
	Sakamoto et al., 2013 (60)	-0.84	-1.70	0.01
	Sung et al., 2012 (64)	-0.93	-1.82	-0.03
Depression	Baker et al., 2022 (42)	-0.61	-1.16	-0.07
	Ceccato et al., 2012 (43)	-0.65	-1.16	-0.14
	Cheng et al., 2012 (45)	-0.57	-1.10	-0.05
	Chu et al., 2014 (47)	-0.56	-1.13	0.01
	Cooke et al., 2010a (48)	-0.64	-1.16	-0.13
	Guétin et al., 2009 (50)	-0.58	-1.11	-0.05
	Huang et al., 2019 (52)	-0.42	-0.88	0.04
	Liu et al., 2021 (54)	-0.60	-1.14	-0.07
	Park et al., 2020 (56)	-0.62	-1.14	-0.11
	Xu et al., 2024 (65)	-0.43	-0.85	0.01
Agitation	Ceccato et al., 2012 (43)	-0.12	-0.29	0.05
	Chen and Pei, 2018 (44)	-0.10	-0.27	0.06
	Cheung et al., 2020 (46)	-0.11	-0.29	0.06
	Cooke et al., 2010b (49)	-0.09	-0.26	0.07
	Lin et al., 2011 (53)	-0.06	-0.23	0.11
	Park et al., 2020 (56)	-0.11	-0.27	0.05
	Ridder et al., 2013 (59)	-0.08	-0.24	0.09
	Sing et al., 2019 (61)	-0.08	-0.25	0.09
	Sung et al., 2006a (62)	-0.04	-0.21	0.12
	Sung et al., 2006b (63)	-0.05	-0.22	0.11
	Sung et al., 2012 (64)	-0.14	-0.31	0.03

SMD, Standardized mean difference; CI, Confidence interval; BPSD, Behavioral and Psychological Symptoms of Dementia.

### 3.6 Publication bias and certainty of evidence

**Figure 7:**
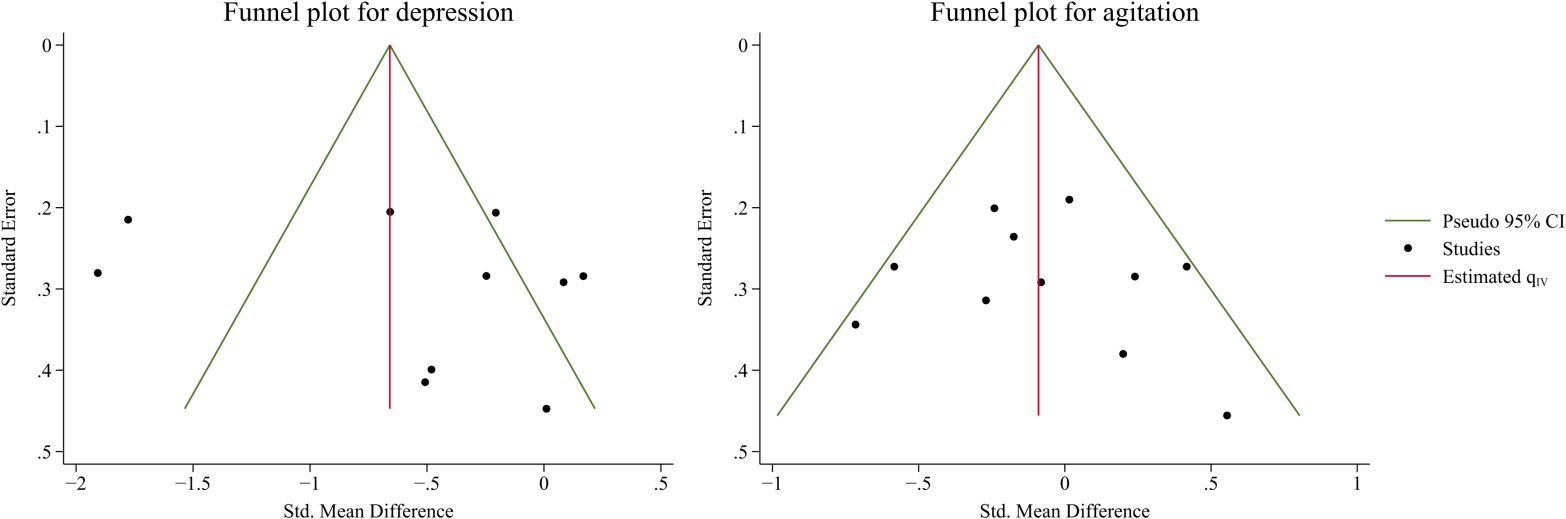
Publication bias for depression (left) and agitation (right).

Given the number of included trials with pooled results for depression and agitation, publication bias was assessed using funnel plots and the Egger’s test. Funnel plots for depression and agitation are presented symmetrically in **Figure 7**. The *p*-values of Egger’s test for depression and agitation were 0.42 and 0.55, respectively, indicating that publication bias did not affect this type of study. According to the GRADE ratings, the certainty of evidence across the outcomes ranged from low to very low, owing to inconsistency, indirectness, imprecision, and publication bias (see **Table 6**).

**Table 6:** Certainty of evidence rating (GRADE).

Outcome	Risk of bias	Inconsistency	Indirectness	Imprecision	Publication bias	Certainty of the evidence
BPSD	No downgrade, as trials at high risk of bias constituted less than 30%.	Downgrade by one level due to unexplained inconsistency (*I^2^* = 43.68%).	Downgrade by one level due to the inclusion of certain participants (OPWD).	No concerns (SMD = -0.33; 95% CI -0.49 to -0.16).	/	⊕⊕⊝⊝ Low
Anxiety	No downgrade, as trials at high risk of bias constituted less than 30%.	Downgrade by two levels due to unexplained inconsistency (*I^2^* = 83.69%).	Downgrade by one level due to the inclusion of certain participants (OPWD).	No concerns (SMD = -0.82; 95% CI -1.53 to -0.10).	/	⊕⊝⊝⊝ Very low
Depression	No downgrade, as trials at high risk of bias constituted less than 30%.	Downgrade by two levels due to unexplained inconsistency (*I^2^* = 87.39%).	Downgrade by one level due to the inclusion of certain participants (OPWD).	No concerns (SMD = -0.57; 95% CI -1.06 to -0.08).	No publication bias is suspected.	⊕⊝⊝⊝ Very low
Agitation	No downgrade, as trials at high risk of bias constituted less than 30%.	Downgrade by one level due to unexplained inconsistency (*I^2^* = 34.66%).	Downgrade by one level due to the inclusion of certain participants (OPWD).	Some concerns (SMD = -0.09; 95% CI -0.25 to 0.07). Downgrade by one level.	No publication bias is suspected.	⊕⊝⊝⊝ Very low

SMD, Standardized mean difference; CI, Confidence interval; *I^2^*, Heterogeneity index in percentage (range: 0-100%); BPSD, Behavioral and Psychological Symptoms of Dementia; OPWD, Older People with Dementia.

## 4 Discussion

Previous studies have predominantly investigated the efficacy of single interventions for OPWD and have provided inconsistent evidence regarding various outcomes (23–33). This study aimed to evaluate the potential multidomain benefits of MBTs for BPSD in OPWD. The pooled results of the meta-analyses indicated that MBTs effectively improved BPSD (SMD = -0.33; 95% CI -0.49 to -0.16; *p* < 0.01), anxiety (SMD = -0.82; 95% CI -1.53 to -0.10; *p* = 0.02), and depression (SMD = -0.57; 95% CI -1.06 to -0.08; *p* = 0.02), with no significant improvements observed in agitation (SMD = -0.09; 95% CI -0.25 to 0.07; *p* = 0.27) among patients with dementia.

Music-based interventions are likely the most common MBT for alleviating BPSD in OPWD, as evidenced in 26 of the 35 trials. Previous studies have confirmed the effectiveness of music-based interventions, particularly in improving anxiety and depression (77–81). Music-based interventions are among the most effective approaches for managing BPSD in OPWD. However, a recent systematic review revealed that music-based interventions may not have achieved the anticipated efficacy, suggesting that the generalizability and applicability of this evidence require further examination (82). Overall, MBTs have significant therapeutic benefits in alleviating BPSD in OPWD, and the underlying mechanisms may be explained from several perspectives. Dementia is associated with the hypothalamus-pituitary-adrenal (HPA) axis and the autonomic nervous system (ANS), which are the main components of emotional regulation (83, 84). By reducing the HPA axis activation and reactivity, MBTs may alleviate the effects of stress and foster multiple positive downstream effects by shifting the ANS balance from primarily sympathetic to parasympathetic, leading to positive changes in cardiac vagal function, mood, energy state, and related neuroendocrine, metabolic, and inflammatory responses (85–89), which may play a potential role in mediating BPSD in OPWD. In addition, an increase in neurotransmitters such as serotonin and dopamine (responsible for regulating emotions and behavior) can be induced through exercise interventions included in MBTs (e.g., Tai Chi, Qigong, and Yoga), which help improve BPSD in OPWD (90).

BPSD represents a heterogeneous group of non-cognitive symptoms and behaviors that occur in patients with dementia, including anxiety, depression, agitation, irritability, apathy, and delusions (91–93). Therefore, in the quantitative analysis, BPSD was subdivided based on existing data to further clarify the efficacy of MBTs for these specific symptoms. Although the pooled results of the meta-analyses indicated that MBTs effectively improved BPSD in OPWD, this impact did not include all symptoms. For instance, according to the pooled results for agitation in OPWD, MBTs did not provide significant therapeutic benefits as previously assumed, which is consistent with previous evidence (30, 32). This may be because, for OPWD, especially those with advanced dementia, the inability to perform activities of daily living, often accompanied by clinical complications such as dysphagia and infections (14), presents a significant challenge in addressing behavioral disorders (including agitation and aggressive tendencies) with short-term MBTs. Finally, in terms of improving anxiety and depression in OPWD, evidence confirming the therapeutic benefits of MBTs is urgently required because of the significant heterogeneity or sensitivity to study selection in the pooled results. This may stem from the fact that the specific interventions, implementation steps, and duration of the interventions varied between studies, resulting in large differences in effect sizes. Additionally, the CIs for certain pooled results were near the threshold of statistical significance after excluding individual studies. This indicates that the sample size and experimental design need to be further increased and refined to more precisely evaluate the potential benefits of MBTs on these outcomes.

Although MBTs are promising complementary therapies for enhancing physical and mental health in OPWD, their differential efficacy should be further explored and validated. Most existing trials focus on the efficacy of a single intervention for BPSD in OPWD while ignoring the differential efficacy between various interventions. This may lead to an overestimation of the actual efficacy of MBTs in clinical practice. In the context of limited medical resources and the increasing prevalence of dementia, MBTs that can be effectively applied to clinical practice should be identified. Doctors and nurses are inclined to use non-pharmacological treatments more than pharmacological treatments in clinical practice (94). Therefore, comparisons between non-pharmacological treatments should receive more attention. A network meta-analysis suggests that individualized nursing, behavior therapy, and reminiscence therapy have significant potential in improving BPSD in OPWD, particularly in reducing anxiety and depression (95). Thus, the differential efficacy of non-pharmacological treatments in improving various aspects of BPSD should be further examined to maximize the clinical benefits of non-pharmacological treatments in the context of limited medical resources. Notably, the efficacy of MBTs in improving BPSD in OPWD may vary depending on the intervention period, frequency, and duration, suggesting that the dose-response relationship of MBTs in this regard is also an important area for investigation. Although this study did not reveal the optimal dosage of MBTs for improving BPSD in OPWD, existing research indicates that the intervention period, frequency, and duration are critical factors influencing efficacy (31, 33, 96). Short-term, high-frequency interventions may lead to faster clinical improvements to some extent, while long-term, low-frequency interventions may help maintain long-term efficacy. Therefore, more high-quality RCTs must be conducted to explore the dose-response relationship of MBTs in improving BPSD in OPWD, and intervention programs should be flexibly adjusted in clinical practice based on patients’ individual differences to achieve the best therapeutic outcomes.

## 5 Limitations

The findings of this systematic review and meta-analysis should be interpreted in the context of these limitations. First, owing to the limited information available on the study population from existing trials, this study mainly examined the overall therapeutic benefit of MBTs for OPWD and did not make specific distinctions based on the type and course of dementia. Second, although there was significant heterogeneity in the pooled results for some outcomes, sources of heterogeneity were not identified because of study data limitations. To address this problem, more comprehensive data should be collected in future studies. Finally, for some outcomes such as anxiety and depression, the pooled results were sensitive to study selection and were less robust; thus, the findings should be interpreted cautiously.

## 6 Conclusions

Effective nursing for patients with dementia is vital, as they are undergoing a major transition in their physical and mental health. Although MBTs effectively improved BPSD in OPWD to some extent, the dose-response relationship of MBTs in improving various aspects of BPSD remains unclear, and these relationships may be influenced by the intervention period, frequency, and duration. In clinical practice, healthcare and social care therapists should develop personalized intervention programs based on patient individual differences and the actual dose-response relationship, which will help maximize the clinical benefits of non-pharmacological treatments in the context of limited medical resources.

## Data Availability

The original contributions presented in the study are included in the article/supplementary material. Further inquiries can be directed to the corresponding author.
